# The impact of canonical Wnt transcriptional repressors TLE3 and TLE4 on postsynaptic transcription at the neuromuscular junction

**DOI:** 10.3389/fnmol.2024.1360368

**Published:** 2024-03-27

**Authors:** Lea Gessler, Danyil Huraskin, Nane Eiber, Said Hashemolhosseini

**Affiliations:** ^1^Institute of Biochemistry, Medical Faculty, Friedrich-Alexander-University of Erlangen-Nürnberg, Erlangen, Germany; ^2^Muscle Research Center, Friedrich-Alexander-University of Erlangen-Nürnberg, Erlangen, Germany

**Keywords:** β-catenin, Axin2, TLE3, TLE4, synaptic gene expression, neuromuscular junction

## Abstract

Here, we investigated the role of the canonical Wnt signaling pathway transcriptional regulators at the neuromuscular junction. Upon applying a denervation paradigm, the transcription levels of *Ctnnb1*, *Tcf7l1*, *Tle1*, *Tle2*, *Tle3*, and *Tle4* were significantly downregulated. A significant decrease in canonical Wnt signaling activity was observed using the denervation paradigm in Axin2-lacZ reporter mice. Alterations in the transcriptional profile of the myogenic lineage in response to agrin (AGRN) suggested that TLE3 and TLE4, family members of groucho transducin-like enhancer of split 3 (TLE3), transcriptional repressors known to antagonize T cell factor/lymphoid enhancer factor (TCF)-mediated target gene activation, could be important regulators of canonical Wnt signaling activity at the postsynapse. Knockouts of these genes using CRISPR/Cas9 gene editing in primary skeletal muscle stem cells, called satellite cells, led to decreased AGRN-dependent acetylcholine receptor (CHRN) clustering and reduced synaptic gene transcription upon differentiation of these cells. Overall, our findings demonstrate that TLE3 and TLE4 participate in diminishing canonical Wnt signaling activity, supporting transcription of synaptic genes and CHRN clustering at the neuromuscular junction.

## Introduction

Wnt glycoproteins are involved in myogenesis and, through both canonical and noncanonical Wnt pathways, regulate muscle formation and maintenance of adult tissue homeostasis (Girardi and Le Grand, 2018). In canonical Wnt signaling, which requires CTNNB1 (β-catenin), the Wnt glycoproteins bind to FZD (Frizzled) and low density lipoprotein receptor-related protein (LRP) receptor complex, thereby leading to the inactivation of GSK3B (glycogen synthase kinase 3β) through DVL (dishevelled). In the absence of Wnt stimulation, CTNNB1 forms a destruction complex with APC (adenomatosis polyposis coli), AXIN1/AXIN2 and GSK3B (MacDonald et al., 2009). Phosphorylation of CTNNB1 by CK1 (casein kinase I) and GSK3B causes ubiquitylation and proteasome mediated degradation of CTNNB1. Wnt stimulation results in the activation of DVL, which leads to phosphorylation dependent recruitment of AXIN1/AXIN2 to the LRP5/6 receptor and disassembly of the CTNNB1 destruction complex. Stabilized CTNNB1 accumulates in the cytoplasm and translocates to the nucleus. There, it complexes with T cell factor/lymphoid enhancer factor (TCF/LEF) transcription factors and acts as a transcriptional coactivator to induce the context-dependent expression of canonical Wnt target genes (Eastman and Grosschedl, 1999).

Four transcription factors mediate CTNNB1 dependent transcription downstream of Wnt signaling, namely TCF7, TCF7L1, TCF7L2 and LEF1 (Cadigan and Waterman, 2012). They all possess a high-mobility group DNA-binding domain with which they interact with specific DNA sequences called Wnt response elements (Arce et al., 2006). TCF/LEF have two regulatory modes (Cinnamon and Paroush, 2008). In the Wnt OFF state TCF/LEF transcriptional activity is blocked by their association to Groucho (GRO)/Transducin-Like Enhancer of split (TLE) transcriptional repressors. In the Wnt ON state nuclear CTNNB1 displaces TLE and facilitates formation of multimeric complexes with transcriptional coactivators which leads to widespread chromatin opening and accessibility. Nevertheless, CTNNB1/TCF complexes can also actively repress transcription of certain genes by recruitment of co-repressors, such as TLEs, histone deacetylases, or competitively displace other transcriptional activators (MacDonald et al., 2009). In mammals, the Tle gene family of transcriptional repressors encodes four members (Tle1-4). They are broadly expressed and involved in the regulation of a variety of developmental processes where they mediate transcriptional repression by physical interaction with transcription factors and recruitment of histone deacetylases (Gasperowicz and Otto, 2005). However, the role of TLEs in myogenesis has not been well studied and, to our knowledge, no evidence has ever been reported for their involvement at neuromuscular junctions (NMJs). Despite cell- and tissue-type specific expression patterns, all mammalian TLEs can interact and repress transcriptional activities of TCF/LEF transcription factors, arguing for a certain redundancy (Brantjes et al., 2001). Axin2 is a direct target of TCF/LEF mediated transcription (Jho et al., 2002; Leung et al., 2002; Lustig et al., 2002) that is expressed in several tissues where Wnt signaling is active (Logan and Nusse, 2004). AXIN2 is widely used as a reporter for canonical Wnt signaling activity.

Previously, our lab demonstrate that AXIN2 and YAP1/TAZ-TEAD signaling members are coexpressed in adult skeletal muscle fibers and canonical WNT proteins concomitantly stimulate both canonical Wnt signaling as well as *Axin2* expression and YAP1/TAZ-TEAD signaling activity during muscle cell differentiation to regulate myotube formation (Huraskin et al., 2016). Recently, our lab used conditional knockout mice for Lrp5 or Lrp6 to investigate their function in muscle cells. While the conditional double knockout mice do not survive beyond E13, the phenotypes of single conditional knockout mice suggest a significantly different role for each of the two receptors, providing an alternative perspective on the participation of the canonical Wnt signaling pathway in adult skeletal muscle cells (Gessler et al., 2022). Very recently, our lab reported on the direct involvement of YAP1 and TAZ in postsynaptic gene expression (Gessler et al., 2023).

At the NMJ several signaling pathways are responsible to ensure clustering of CHRNs (nicotinic acetylcholine receptors, AChRs) at the postsynaptic apparatus (Li et al., 2018). A neural isoform of a large heparansulfate proteoglycan, called AGRN (agrin), is released by the nerve ending and involved in both stabilization of clusters of existing acetylcholine receptors and stimulation of synaptic gene expression. AGRN interacts with its receptor LRP4 and thereby activates the co-receptor MUSK (MuSK), a muscle-specific receptor tyrosine kinase. The clustering of CHRNs serves as a hallmark for the presence of the postsynaptic apparatus within the endplate zone, the central part of each muscle fiber (Li et al., 2018).

Muscular CTNNB1 gain-of-function phenotype is associated with presynaptic defects *in vivo* resulting from changed neuromuscular retrograde signaling (Li et al., 2008; Liu et al., 2012; Wu et al., 2015), however, CTNNB1 loss of function also affects CHRN cluster size and distribution (Li et al., 2008). In cultured muscle cells CTNNB1 exerts both positive and negative regulation of CHRN clustering, by either acting cytosolic as a link between RAPSN (Rapsyn), a peripheral membrane protein required for CHRN clustering at NMJs, and the cytoskeleton or negatively by regulating *Rapsn* expression in the nucleus, respectively (Zhang et al., 2007; Wang et al., 2008). However, the downregulation of *Rapsn* transcription by CTNNB1 has been found to be TCF independent (Wang et al., 2008). On the other hand, β-galactosidase reporter was accumulated in synaptic nuclei in muscle fibers of Axin2-lacZ reporter mice (Huraskin et al., 2016), suggesting that canonical Wnt signaling, and TCF/LEF target gene expression are active at the NMJs. Similarly, in X-Gal stained TCF/LEF-lacZ reporter mouse muscles a pronounced neuromuscular signal is detectable (Kuroda et al., 2013).

In the present study, we identified a loss of canonical Wnt signaling activity upon denervation and wondered whether and which TLE transcriptional repressors of canonical Wnt signaling are expressed in differentiated muscle cells and whether and how they are involved in transcriptional regulation of postsynaptic gene expression.

## Materials and methods

### Plasmids, primers, *in situ* probes

For CRISPR/Cas9 mediated gene editing guide sequences (spacers) were designed with the online tool E-CRISP (Heigwer et al., 2014). Search parameters were set to medium stringency and sequences were chosen, that were exonal and closest to the ATG in 3′ direction. Twenty bp long complementary oligonucleotide pairs with overhangs were cloned into the pX330-U6-Chimeric_BB-CBh-hSpCas9 vector from Feng Zhang Lab (Addgene plasmid # 42230). Cloning procedure was based on a published protocol (Cong et al., 2013) and available online at: https://media.addgene.org/cms/filer_public/e6/5a/e65a9ef8-c8ac-4f88-98da-3b7d7960394c/zhang-lab-general-cloning-protocol.pdf. Plasmids were transformed in NEB 5-α *E. coli* bacteria (New England Biolabs, C2987), extracted from bacteria by alkaline lysis with the Nucleobond PC100 Midiprep Kit (Macherey-Nagel, 740573) and verified by restriction digestion and sequencing.

For generation of *in situ* riboprobes, corresponding regions were amplified from mouse muscle 1st-strand cDNA using the same primers as for the quantification of respective transcripts in qPCR studies (Supplementary Table S1) and ligated into EcoRV digested pBluescript SK(+) plasmid. Directionality and correct sequence of the insert was verified by DNA sequencing. Riboprobes were made by linearization of the plasmid and transcription with the T7 RNA polymerase.

pMAX-GFP (Lonza, VPD-1001) was used as control for transfection.

### *In situ* hybridization, RNA extraction, reverse transcription, PCR

For *in situ* hybridization experiments, newborn wild type pups were decapitated immediately after birth and diaphragm was dissected and fixed overnight in 4% paraformaldehyde (PFA) and dehydrated in gradient of 25 to 100% methanol solutions for 15 min each. Diaphragms were stored at −20°C. To perform *in situ* hybridization diaphragm were rehydrated, quickly washed in PBST and treated for 15 min with Proteinase K (20 μg/mL). After refixation in 0.2% glutaraldehyde in 4% PFA, diaphragms were washed, incubated for 2 h in pre-hybridization buffer and hybridized overnight at 55°C with corresponding denatured (5 min 95°C, followed by 3 min on ice) riboprobes (10 μL/mL). Next day diaphragms were washed, blocked with 10% FCS in TBST and incubated for 4 h with a 1:2,000 dilution of the anti-Digoxigenin-AP antibody (Roche Diagnostics, 11093274910) in 1% FCS in TBST. After six washing steps, diaphragms were kept in TBST overnight. Next day diaphragms were equilibrated in NTM solution (0.1 M Tris, pH 9.5, 0.5 M NaCl, 0.05 M MgCl_2_, 0.1% Tween 20) and developed with 90 mM NBT (Roche Diagnostics, 11383213001) and 110 mM BCIP (Roche Diagnostics, 11383221001) in NTM solution.

Total RNA was extracted from primary muscle cells or hindlimb muscles of adult mice with TRIzol reagent (Thermo Fisher Scientific, 15596026) (Cheusova et al., 2006) and reverse transcribed with M-MuLV Reverse Transcriptase (New England Biolabs, M0253) according to the manufacturer’s instructions. cDNAs were used with mouse-specific primers (Supplementary Table S1) for quantitative PCR reactions using the PowerUp SYBR Green Master Mix (Thermo Fisher Scientific, A25743) and the C1000 Thermal Cycler with the CFX96 Real-Time PCR Detection System (Bio-Rad) according to the manufacturer’s instructions. After the PCR run, sizes of amplified DNA products were verified by agarose gel electrophoresis. The obtained Ct values were analyzed using GraphPad Prims software. Ct values of the genes of interest were normalized to Ct values of the internal control (Rpl8 gene) and related to the control sample (fold change = 2^−ΔΔCt^) (Livak and Schmittgen, 2001; Schmittgen and Livak, 2008).

### Tissue culture, culturing of primary muscle cells, transfection, generation of CRISPR/Cas9 knockout cells

Primary skeletal muscle satellite cells were prepared from muscles of 2–3 months old adult C57BL/6 wild type mice using the mouse Skeletal Muscle Dissociation Kit (Miltenyi Biotech, 130-098-305), followed by mouse MACS Satellite Cell Isolation Kit (Miltenyi Biotech, 130-104-268). Cells were used for immediate RNA extraction or seeded on Matrigel-coated plates (Thermo Fisher Scientific, CB-40234) in growth medium [40% DMEM, 40% Ham’s F10, 20% FCS, 1% penicillin/streptomycin, and recombinant human fibroblast growth factor (Promega, G507A, 5 ng/mL)]. To yield sufficient total RNA amounts from directly isolated muscle satellite cells for cDNA synthesis, a total of 8 g of mouse muscle tissue was used for isolation and the cells were pooled before RNA extraction. For differentiation to myotubes, primary skeletal muscle cells were grown to confluency and cultured in differentiation medium (95% DMEM, 5% horse serum, 1% penicillin/streptomycin).

For CRISPR/Cas9-mediated generation of knockout cells, guide sequences were designed to target the coding sequence closest to the start codon of the gene of interest to hit as many splice variants as possible, and were cloned into pX330-U6-Chimeric_BB-CBh-hSpCas9 vector (Cong et al., 2013). Twenty-four hours after co-transfection of the respective vectors and a GFP expressing plasmid into purified primary wild type muscle satellite cells with a low passage number (less than 3), single cells were detached and single-cell FACS sorted onto Matrigel-coated 96 Well plates containing growth medium and clonally expanded. After clonal expansion, several independent correct knockouts for *Tle3* and *Tle4* were identified by absence of protein of interest by immunofluorescence microscopy and western blot. Clones of each gene knockout were sequenced to confirm and identify the genomic bi-allelic mutations. For this, a region with about 100–150 bps flanking each side of the target site was amplified and the PCR products were sequenced with one of the primers (Supplementary Table S1). The Degenerate Sequence Decoding strategy (Ma et al., 2015) was employed to decode sequences of each allele from overlapping peaks of sequencing chromatograms. Clones with undetectable protein of interest consistently featured indels in the vicinity of the target sequence. Only clones with frameshift mutations causing premature stops on both alleles were used for further studies.

To induce clustering of CHRNs, cells were seeded onto 0.1% gelatine (Thermo Fisher Scientific, Cascade Biologics Attachment Factor 1x, S-006-100) coated dishes, differentiated to myotubes for 6 days and treated with neural AGRN-conditioned media. The production of AGRN-conditioned media was described previously (Kroger, 1997). AGRN-conditioned medium was added at 1:8 dilution to myotubes. CHRN clusters were detected and quantified 16 h later, as described below.

### Protein lysates, SDS-PAGE, western blot

To obtain cytosolic and nuclear fractions of protein, cells were lysed in ice cold protein lysis buffer A (10 mM HEPES pH 7.9, 10 mM KCl, 0.2 mM EDTA, 2 mM DTT, 20 μg/mL Aprotinin and 20 μg/mL Leupeptin in deionized water) and scraped off with a cell scraper into a reaction tube. After 5 min on ice, 1% NP-40 was added and vortexed for 10 s. The lysate was centrifuged for 30 s at 16.000 × g to acquire the supernatant containing the cytosolic protein fraction. The pellet was resuspended in the same lysis buffer with additional 400 mM NaCl and 1% NP-40 and rotated for 15 min at 4°C before centrifugation for 5 min at 16.000 × g to acquire the nuclear protein fraction in the supernatant. Whole cell extracts were prepared by scraping the cells into protein lysis buffer A, incubating on ice for 5 min and addition of 1% NP-40 before vortexing for 10 s. Then 400 mM NaCl was added, and the lysate was rotated for 15 min at 4°C. After centrifugation for 5 min at 16.000 × g the supernatant containing the whole cell protein extract was used. All protein lysates were diluted with Laemmli buffer, boiled at 95°C for 5 min, and separated by sodium dodecyl sulfate (SDS) polyacrylamide gel electrophoresis with the Biometra Minigel Twin system. Separated proteins were blotted on to a nitrocellulose membrane (Sigma Aldrich, Protran BA 85), blocked in 5% BSA or 5% non-fat dry milk in PBS or TBS with 0.1% Tween20 slowly shaking for 1 h at room temperature.

After blocking the membranes were incubated with primary antibodies at 1:3,000 dilution slowly shaking over night at 4°C: TLE3 (Santa Cruz Biotechnology, sc-514798), TLE4 (Santa Cruz Biotechnology, sc-365406), beta-Catenin (Cell Signaling, 9582), GAPDH (Santa Cruz Biotechnology, sc-25778). Corresponding HRP-linked secondary antibodies against rabbit or mouse (Cell Signaling, 7074, 7076) at a 1:3,000 dilution were bound for 2 h at room temperature. Protein bands were detected with chemiluminescence reagent solution and protein bands were exposed on RXSuper X-Ray films (Fuji Medical). The chemiluminescence reagent consisted of 3 mL of 0.25 mg/mL Luminol (Sigma Aldrich, A-4685) in 0.1 M Tris pH 8.6 solution and 40 μL of 1.1 mg/mL Para-hydroxy-cumarinic acid (Sigma Aldrich, C-9008) in DMSO, mixed with 3 mL of 1XPBS and 1.2 μL of 30% H_2_O_2_. For difficult-to-detect proteins, Amersham ECL Advance Western blotting Detection Kit was used instead (GE Healthcare, RPN2135) according to manufacturer’s instructions. Western blot results were quantified by densitometric analysis using Fiji image processing package^1^ (Schindelin et al., 2012). Films were scanned with an Epson Expression 1600 Pro Scanner at 300 dpi. After background subtraction, protein bands of interest were labeled and measured. For quantification the protein band intensity was normalized to intensity of GAPDH protein band of the respective sample.

### X-Gal staining, immunofluorescence staining, fluorescence microscopy

X-Gal stainings on muscle tissues were performed as described previously (Huraskin et al., 2016). Stainings were documented using a Zeiss Axio Examiner Z1 microscope (Carl Zeiss MicroImaging) equipped with an AxioCam MRm camera (Carl Zeiss MicroImaging) and ZEISS AxioVision Release 4.8 (Carl Zeiss MicroImaging) (Eiber et al., 2019).

For immunofluorescence analysis cells were fixed in 2% PFA for 15 min on ice, permeabilized for 10 min in 0.1% Triton X-100 in PBS, blocked in 10%FCS (v/v), 1%BSA (v/v) in PBS for 1 h at room temperature and incubated with antibodies at 1:1,000 dilution at 4°C overnight. Secondary antibodies conjugated to Cy3 or Alexa Fluor 488 immunofluorescent dyes (Dianova, 111-165-144, 115-165-146) were used for detection.

For detection of CHRNs in cells, counterstaining was performed with DAPI to visualize nuclei. To quantify CHRN clusters images of BTX and DAPI stained myotubes were acquired with a 20× objective on a Leica DMI6000B microscope (Leica Microsystems), exported as TIF image files and quantified with Fiji software. A constant threshold was set for all samples to subtract background signal and create a mask for quantification of BTX fluorescence signal intensity with the Analyze Particles function. The normalized BTX fluorescence intensity was calculated as the total raw integrated density of BTX fluorescence signal divided by the number of nuclei in the image. Signals from undifferentiated cells or cell debris were excluded prior to quantification by manual selection.

### Mouse procedures

Mouse experiments were performed in accordance with animal welfare laws and approved by the responsible local committees (animal protection officer, Sachgebiet Tierschutzangelegenheiten, FAU Erlangen-Nürnberg, AZ: I/39/EE006 and TS-07/11), government bodies (Regierung von Unterfranken). Mice were housed in cages that were maintained in a room with temperature 22 ± 1°C and relative humidity 50–60% on a 12 h light/dark cycle. Water and food were provided *ad libitum*. Mouse mating and genotyping were performed as previously described (Yu et al., 2005). All adult muscles which were analyzed in this manuscript commonly belong to animals of 2–3 months of age.

### Statistical analysis

Statistical analysis was performed in GraphPad Prism 10 Software as indicated. Outliers were identified by GraphPad Prism and not used for analysis. Wherever not differently stated, unpaired student’s *t*-test and SD error bars were used. *p*-value format: GraphPad style which reports four digits after the decimal point with a leading zero: ns (not significant) *p* > 0.05, *p ≤ 0.05, **p ≤ 0.01, ***p ≤ 0.001, and ****p ≤ 0.0001.

## Results

### Denervation diminishes canonical Wnt signaling in adult muscle fibers

**Figure 1:**
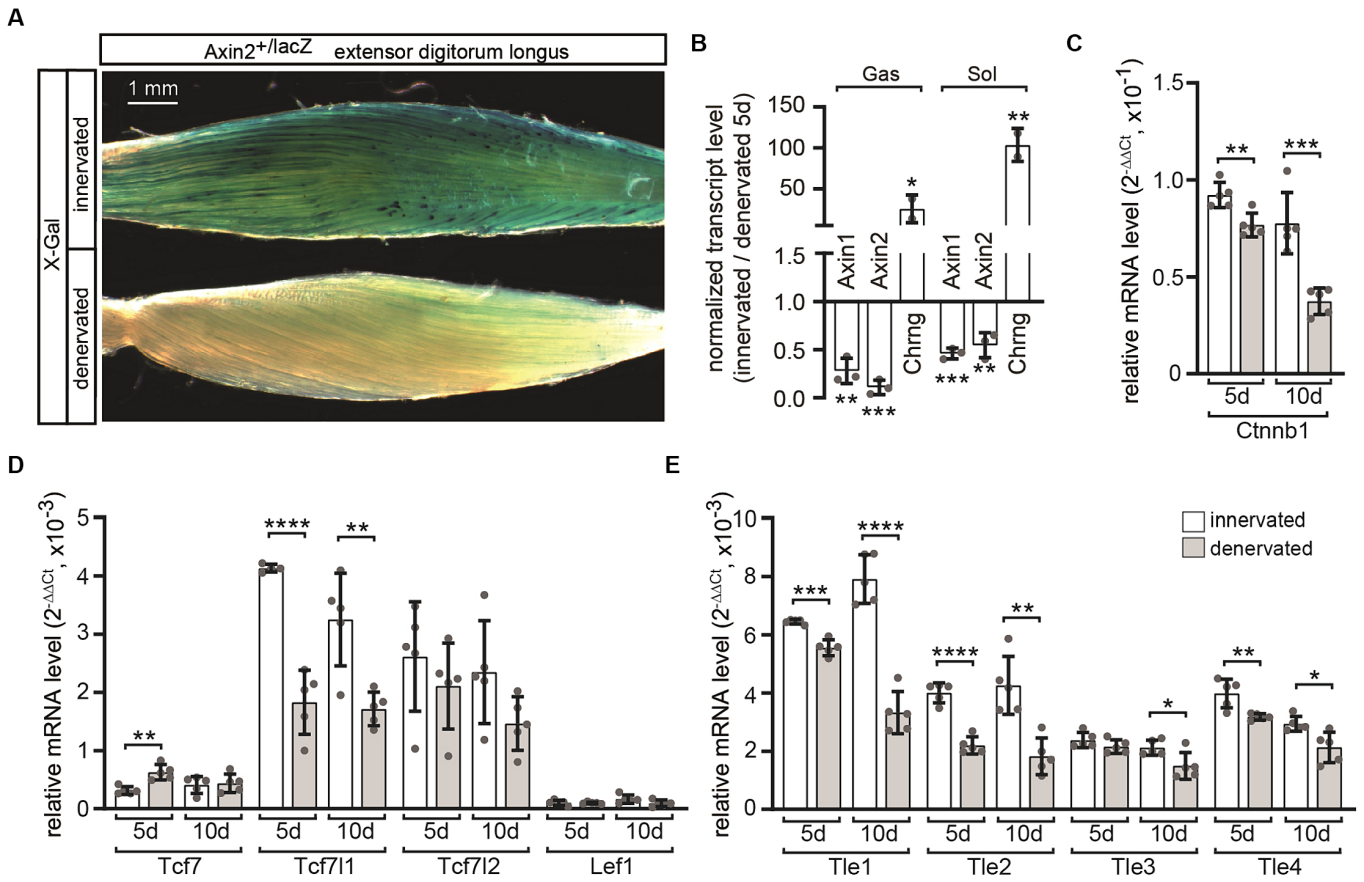
Transcriptional alterations of Wnt and YAP1/TAZ-TEAD signaling pathway constituents in denervated muscle. Axin2^+/lacZ^ mice were subjected to unilateral sciatic nerve lesion and their denervated and contralateral innervated hind limb muscles were used for analysis 5 and 10 days later. **(A)** After 5 days extensor digitorum longus muscles were isolated from denervated and contralateral innervated leg, stained with X-Gal and compared. Typical blue staining of the innervated muscle was almost completely abolished in the denervated contralateral counterpart. **(B)** Five days after denervation gastrocnemius and soleus muscles were used for RNA extraction and transcript amounts of *Chrng*, *Axin1* and *Axin2* were quantified by qPCR. A strong increase of *Chrng* expression proves a successful denervation. Notably both *Axin1* and *Axin2* were downregulated after denervation. The graph has been designed to indicate upward regulation for all numbers above 1 and downward regulation for all numbers below 1; the number “1” indicates no change. Note, asterisks above the bars reflect statistics for innervated vs. denervated. **(C–E)** mRNA level of nuclear effectors of canonical Wnt signaling in gastrocnemius muscles denervated for 5 or 10 days were quantified. **(C)** A significant reduction in *Ctnnb1* expression after denervation could be observed. **(D)** Of the investigated *Tcf*/*Lef* family members, only *Tcf7l1* mRNA levels decreased significantly after denervation. An increase of the *Tcf7* expression levels was detectable after denervation. **(E)** All *Tle* family members were downregulated after denervation. *N* ≥ 3 mice per genotype, each qPCR was performed ≥three times in duplicate. Note the information on the color assignment of the columns in the diagram **(E)**.

Previously, it was shown that β-galactosidase expression *in vivo* in muscle of canonical Wnt reporter mice accumulates at the NMJ (Kuroda et al., 2013; Huraskin et al., 2016), suggesting that motor nerves contribute to canonical Wnt signaling. However, the physical presence of nerve endings at NMJs is not required for postsynaptic gene expression (Duclert and Changeux, 1995), apparently postsynaptic gene transcription is mediated by specific signaling pathways at NMJs. For example, the ectopic expression of AGRN, neural active MUSK or NRG1/ERBB signaling upregulates the transcription of synaptic genes (Meier et al., 1997; Jones et al., 1999; Buonanno and Fischbach, 2001; Moore et al., 2001; Schaeffer et al., 2001). This implies that the continuous presence of the nerve is not required for inducing NMJ-specific transcription but rather important for the repression of CHRN transcription outside the NMJ area. We were interested in how denervation of the muscle would affect canonical Wnt signaling activity at the NMJ. Unilateral sciatic nerve lesions were set in heterozygous Axin2^+/lacZ^ reporter mice and we assessed β-galactosidase reporter expression 5 and 10 days later by X-Gal staining (Figure 1A) and changes in *Axin1* and *Axin2* transcript levels by qPCR (Figure 1B). We found that in denervated extensor digitorum longus muscle the expression of *Axin2*, as shown by blue staining, was completely absent (Figure 1A), confirming the abrogation of canonical Wnt signaling. At the same time, *Axin1* and *Axin2* transcripts were downregulated in denervated soleus and gastrocnemius muscles of Axin2-lacZ mice in comparison to innervated contralateral control muscles (Figure 1B), confirming the inhibition of β-galactosidase reporter expression. As expected and serving as a positive control (Evans et al., 1987), the transcript level of the *Chrng* gene, was strongly upregulated following denervation (Figure 1B). We wanted to investigate if and how expression of transcriptional effectors of canonical Wnt signaling were changed *in vivo* in denervated gastrocnemius muscles 5 or 10 days after sciatic nerve lesion compared to contralateral innervated muscle of the same mouse. In response to denervation, the transcript levels of *Ctnnb1* and *Tcf7l1*, which are transcriptional effectors of the canonical Wnt signaling pathway, as well as the transcriptional repressors *Tle1*, *Tle2*, and *Tle4*, are significantly reduced (Figures 1C–E). Overall, these data suggest that after denervation there is a shift towards a loss of canonical Wnt signaling activity in skeletal muscle fibers.

### Expression profiling of the transcriptional regulators of canonical Wnt signaling during myogenesis

**Figure 2:**
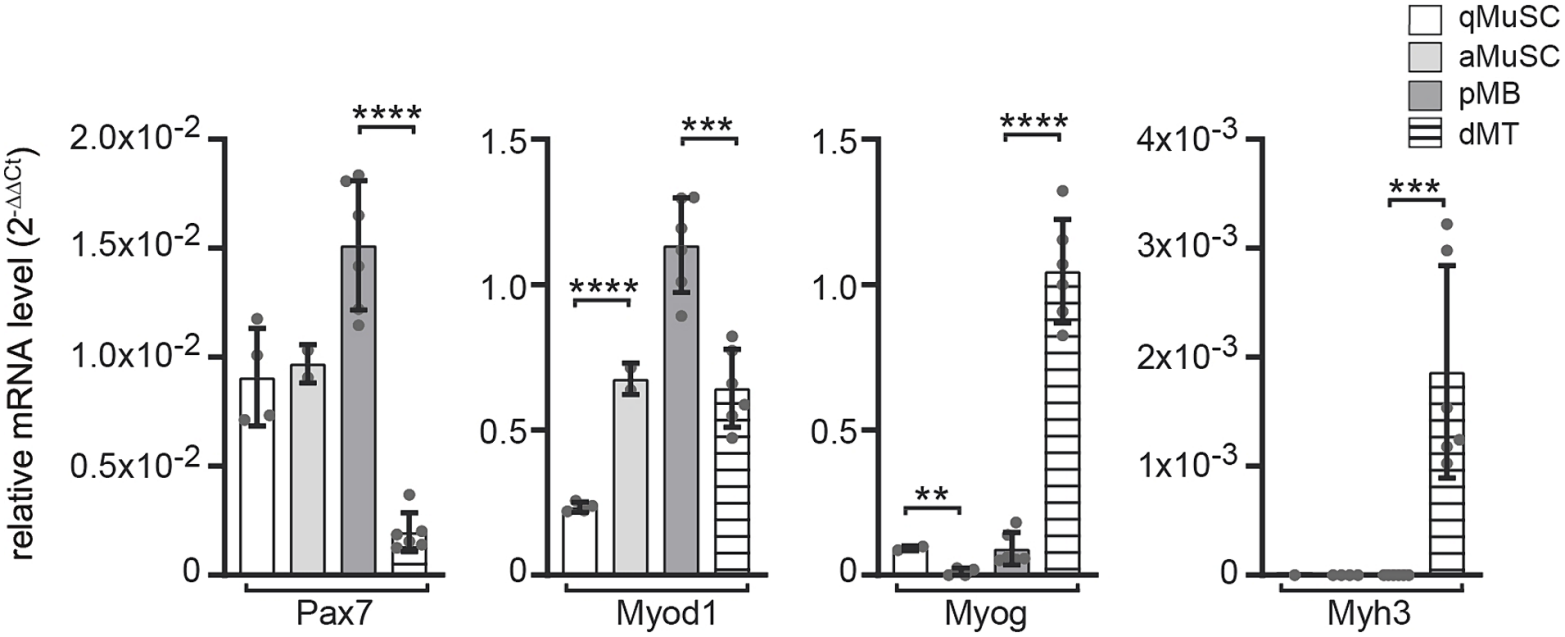
mRNA expression level changes of the typical myogenic markers *Pax7*, *Myod1*, *Myog* and *Myh* in *ex vivo* MuSCs under proliferative and differentiating conditions. mRNA content of wild type primary muscle stem cells (MuSCs) was quantified at different myogenic stages, like (1) qMuSCs, which are satellite cells that are either freshly isolated or plated and in a quiescent state, (2) aMuSCs, which are activated satellite cells that have been cultured for 3 days under proliferative conditions, (3) satellite cell-derived myoblast cultures after five passages under proliferative conditions (referred to as proliferating myoblasts, or pMBs), and (4) after 6 days under differentiating culture conditions, during which many cells fused to form myotubes (referred to as differentiating myotubes, or dMTs). Expression changes of myogenic lineage markers indicate resemblance to different stages of the myogenic lineage: qMuSC (high *Pax7*, low *Myod1*), aMuSC (high *Pax7*, high *Myod1*), pMB (high *Pax7*, high *Myod1*, low *Myog*), dMT (low *Pax7*, high *Myog*, high embryonal *Myh3*). Each qPCR was performed at least three times in duplicate for *N* ≥ 3 sets of cells. Note, the color and pattern assigned to the columns on the right. In addition to unpaired student t test, one-way ANOVA statistics was employed, the *post hoc* method Tukey’s multiple comparisons test was used and ANOVA GraphPad Prism summary results are presented for the different groups: Pax7****, Myod1****, Myog****, Myh3***.

To better understand which transcriptional regulators of the canonical Wnt signaling pathway participate at which stage of the myogenic fate and whether they are involved in postsynaptic gene expression, we analyzed their transcriptional profiles at distinct myogenic stages to screen for those transcriptional regulators which expression was elevated in myotubes. We isolated and quantified RNA from four distinct stages in the myogenic lineage. First, we purified wild type mouse muscle satellite cells (MuSCs) immediately after isolation or after plating; these cells mirror quiescent satellite cells (qMuSCs). Second, we harvested myogenic cells after 3 days in culture under proliferative conditions, likely these are activated satellite cells (aMuSCs). Third, we collected cells from satellite cell-derived myoblast cultures after five passages under proliferative conditions. These cells we considered being proliferating myoblasts (pMBs). And fourth, we collected cells after 6 days under differentiating culture conditions, when many cells fused to myotubes. These cells should reflect differentiating myotubes (dMTs). In the following writing and in the presented figures we point to these four stages using the abbreviations, qMuSC, aMuSC, pMB, and dMT. We assessed the transcriptional profiles of typical myogenic lineage markers *Pax7*, *Myod1*, *Myog* and embryonal *Myh3* (Figure 2). Immediately after isolation from murine skeletal muscles, qMuSCs showed high expression of *Pax7* but low *Myod1* expression. In contrast, RNA samples of aMuSCs showed elevated *Myod1* expression (Figure 2), suggesting activation of MuSCs. Late myogenic markers *Myog* or *Myh3* were not significantly expressed in these samples. The transition from pMBs to dMTs resulted in a decrease in *Pax7* and *Myod1* expression and an increase in the expression of the early myogenic differentiation marker *Myog* and the late myogenic differentiation marker *Myh3* (Figure 2). This confirms that the analyzed samples represent different stages of myogenesis.

**Figure 3:**
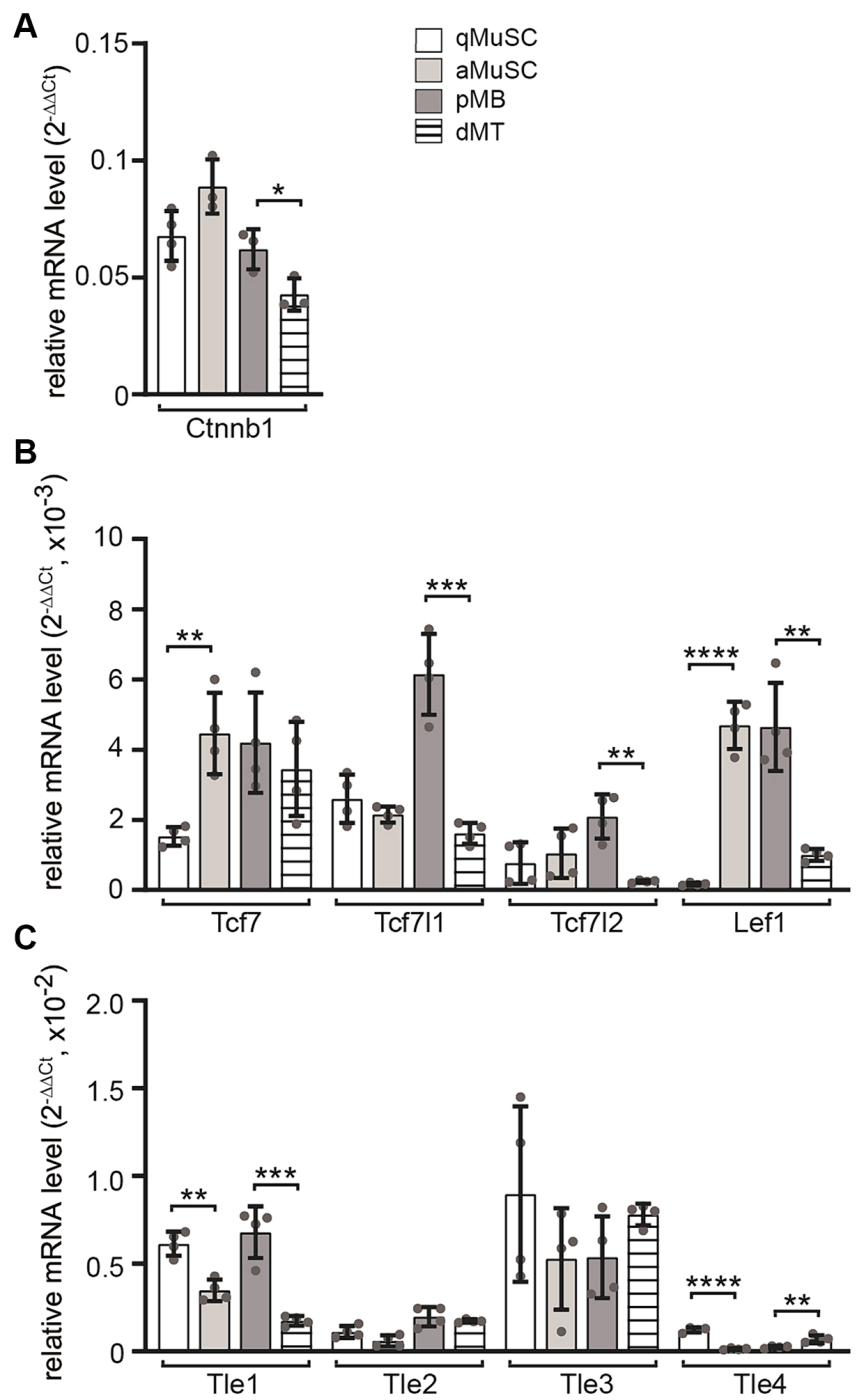
Transcript profiles of members of transcriptional effectors for canonical Wnt signaling in MuSCs were analyzed at four different myogenic lineage stages. The mRNA utilized for experiments shown in Figure 1 was also used to analyze transcript levels of *Tcf* and *Tle* members in qMuSCs, aMuSCs, pMBs, and dMTs. **(A)** *Ctnnb1* transcript levels slightly increased during cell proliferation and significantly decreased upon differentiation. **(B)** Expression changes of *Tcf*/*Lef* family transcription factors were quantified. *Tcf7* and *Lef1* transcript levels increased during proliferation, while most factors were downregulated during differentiation. **(C)** Most members of the *Tle* family exhibit reduced expressed under proliferative conditions. During differentiation, the expression of *Tle1* decreased, while *Tle4* increased. Each qPCR was performed in triplicate across at least three sets of cells. Please note the column color and pattern assignments provided in diagram **(A)**. In addition to unpaired student *t* test, one-way ANOVA statistics was employed, the *post hoc* method Tukey’s multiple comparisons test was used and ANOVA GraphPad Prism summary results are presented for the different groups: Ctnnb1**, Tcf7*, Tcf7l1****, Tcf7l2**, Lef1****, Tle1****, Tle2***, Tle3 not significant, Tle4****.

Next, the transcript levels of *Ctnnb1*, *Tcf*/*Lef* transcription factors and *Gro*/*Tle* transcriptional repressors of canonical Wnt signaling were analyzed at four myogenic stages. During MuSC activation, *Ctnnb1* transcript levels showed a slight upregulation, which was followed by a decrease during differentiation to myotubes (Figure 3A). The transcriptional changes of *Tcf*/*Lef* transcription factors *Tcf7, Tcf7l1, Tcf7l2* and *Lef1* correlated with changes in *Ctnnb1* transcript levels (Figure 3B). Under proliferating conditions, the expression of *Tcf7* and *Lef1* in MuSCs increased significantly, while in myotubes, the expression of *Tcf7l1, Tcf7l2* and *Lef1* decreased significantly (Figure 3B). The expression of *Tle* family transcriptional repressors of Wnt signaling showed an opposite behavior. Under proliferative conditions, both *Tle1* and *Tle4* transcript levels decreased significantly, while under differentiating conditions *Tle1* expression decreased and *Tle4* expression increased (Figure 3C). The transcriptional changes of positive and negative regulators of the canonical Wnt signaling pathway suggest a temporary increase in activity which would decrease again in differentiating myotubes due to the expression of the CTNNB1 inhibitor *Axin2* (Huraskin et al., 2016).

### The clustering of CHRN, which is dependent on neural AGRN, is impaired by the ablation of *Tle4* expression in myotubes, but not by *Tle3* expression

**Figure 4:**
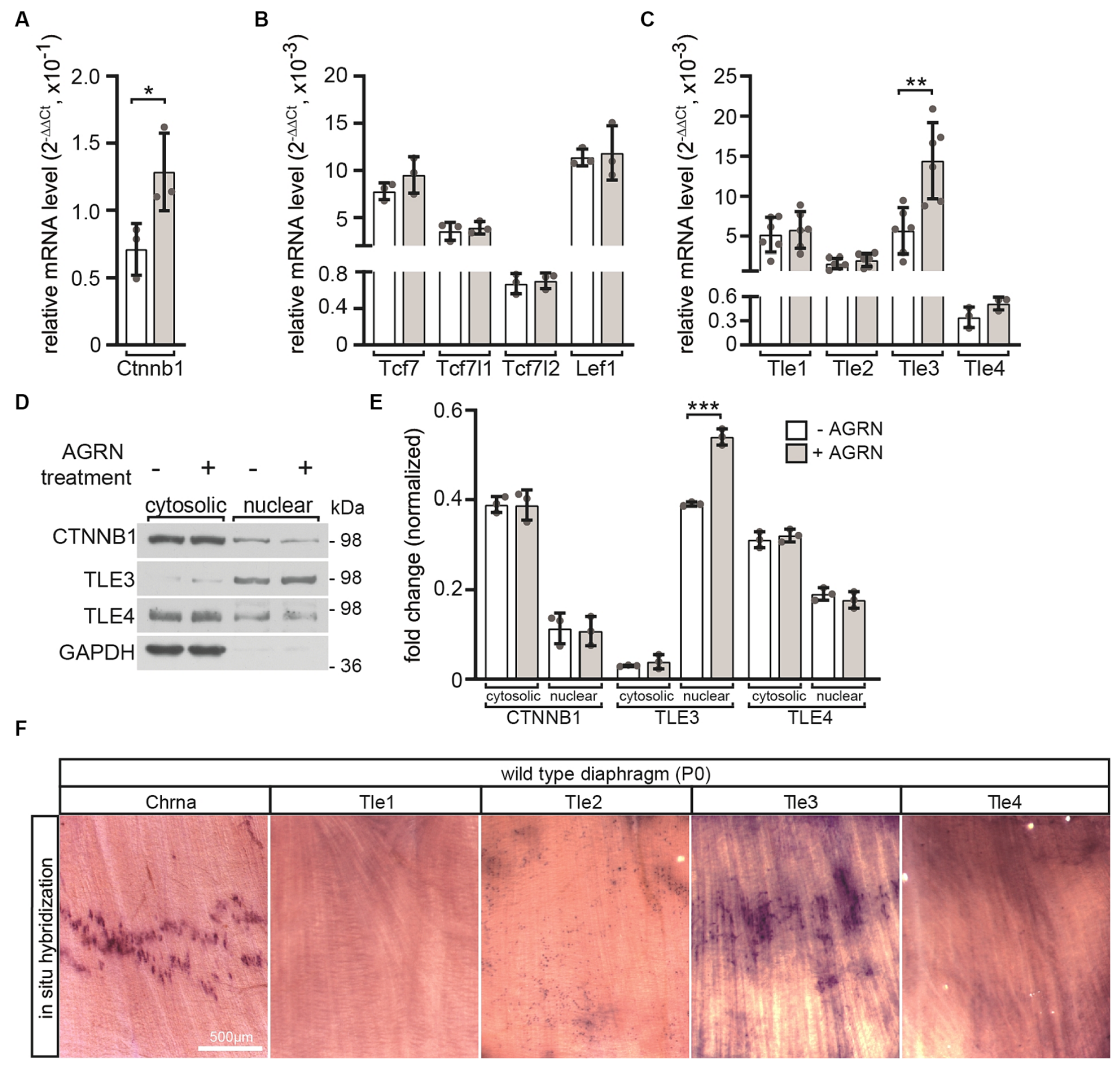
The impact of AGRN-mediated response on transcriptional regulators in cultured primary wild type myotubes. MuSCs were differentiated into myotubes for 3 days. They were then treated with AGRN-conditioned media (Kroger, 1997) for 16 h before mRNA was extracted for quantification **(A–C)**. Alternatively, cells were lysed and cytosolic and nuclear fractions of the protein lysates were analyzed by SDS-PAGE and western blot **(D,E)**. AGRN treatment of myotubes significantly increased expression of *Ctnnb1* **(A)**, *Tle3* and *Tle4* **(C)** in comparison to control cells not incubated with neural AGRN. **(B)** The expression of none of the *Tcf*/*Lef* family members significantly changed in response to AGRN. **(D)** Representative western blots showing cytosolic and nuclear fractions of protein lysates, and the respective quantifications are presented **(E)**. Normalization was performed using GAPDH **(E)**. The uniform loading of all SDS-PAGE lanes was additionally confirmed by Ponceau S staining of the Western blot membrane. **(D,E)** A change in the protein levels or cytosolic/nuclear translocation of CTNNB1 or TLE4 could not be detected. However, after AGRN treatment, there was a significant increase in nuclear TLE3 levels. **(F)** *In situ* hybridization was performed using riboprobes that were complementary to mRNAs of *Chrna1*, *Tle1*, *Tle2*, *Tle3* and *Tle4* mRNAs on neonatal diaphragm muscles. The results show that *Chrna1* and *Tle3* transcripts accumulated in the endplate zone located at the center of the muscle. The experiment was replicated three times in duplicate with at least three sets of cells for each experiment. Note the information on the color assignment of the columns is as presented in the diagram **(E)**.

We investigated which transcriptional regulators of canonical Wnt signaling responded directly to activation of AGRN/LRP4/MUSK signaling with transcriptional and translational changes. After differentiating cultured MuSCs into myotubes, we initiated stimulation of the AGRN/LRP4/MUSK signaling by adding conditioned media containing neural AGRN. Transcript levels of *Ctnnb1* (Figure 4A) and *Tle3* (Figure 4C) significantly increased after AGRN treatment. Transcript levels of other transcriptional regulators, such as TCF/LEF transcription factors, remained unchanged (Figure 4B). This suggests that they eventually do not play a significant role in neuromuscular function, as assumed due to their decreased expression after differentiation into myotubes (Figure 3B). The nuclear protein levels of TLE3 were significantly increased by AGRN treatment (Figures 4D,E), as confirmed by transcriptional changes (Figure 4C). In order to investigate the localization of *Tle3* and *Tle4* transcripts in skeletal muscle fibers *in vivo*, we conducted *in situ* hybridization with riboprobes on diaphragm muscles of newborn wild type mouse pups. Our results showed that *Tle3* transcripts exhibited a significant accumulation in the central area of muscle fibers, which is also the location of the endplate zone containing NMJs, as indicated by *Chrna1* expression (Figure 4F).

**Figure 5:**
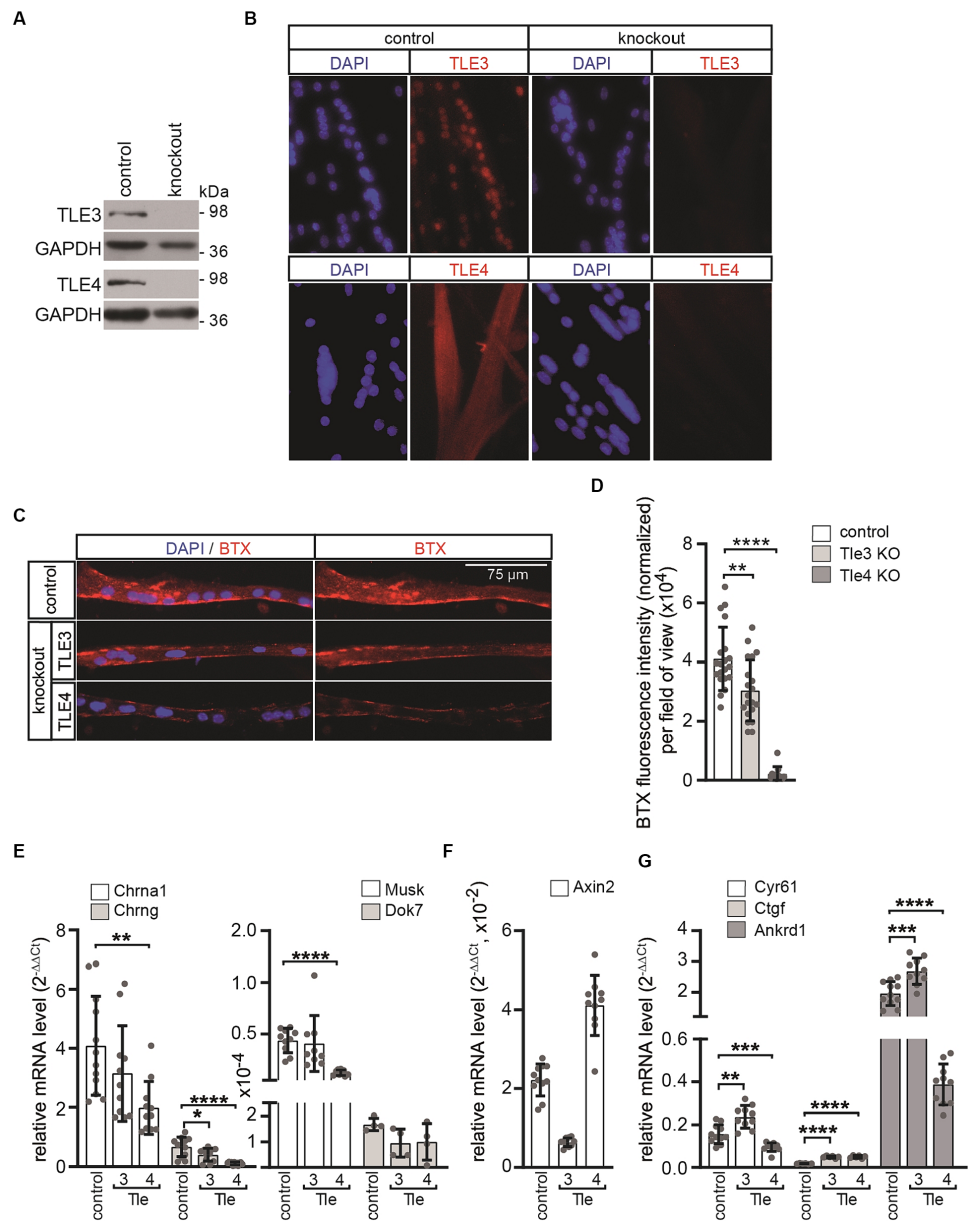
In primary muscle cells differentiated from *Tle3* and *Tle4* knockouts, AGRN-induced CHRN clustering and synaptic gene expression are affected. **(A)** The target proteins TLE3 or TLE4 are not detectable in protein lysates of the respective knockout clones by western blot. **(B)** The absence of the target protein was further verified by immunofluorescence. **(C)** Control and CRISPR knockout MuSCs were differentiated to myotubes for 5 days and treated with AGRN-conditioned media for 16 h before fixation and staining with BTX and DAPI. **(D)** CHRN aggregates on myotubes were quantified with Fiji software processing from 20-fold objective images and normalized to number of nuclei in the myotubes. While *Tle3* deficient cells showed a slight but significant reduction, *Tle4* knockouts showed a severe reduction of CHRN clusters, compared to control, as observed by reduction of BTX fluorescence intensity. *N* ≥ 2 clones per knockout, *N* ≥ 3 sets of cells and *N* ≥ 16 images per set and sample. **(E)** Control and CRISPR knockout MuSCs were differentiated to myotubes for 5 days, RNA was extracted and transcript levels of *Chrna*, *Chrng*, *Musk* and *Dok7* were assessed by qPCR. *Tle4* CRISPR knockout MuSCs exhibited the strongest effect on expression of these genes in comparison to control. **(F)** Same CRISPR knockout MuSCs, like in **(E)**, were analyzed for their profile of *Axin2* transcripts, a typical CTNNB1-dependent target gene. The transcript level of *Axin2* is significantly reduced in *Tle3* knockout MuSCs and upregulated in *Tle4* knockout MuSCs. **(G)** Using the same cells like in **(E)** the profile of common TEAD target genes *Ankrd1*, *Cyr61* and *Ctgf* was investigated. Note, transcription of TEAD target genes shows a specific pattern after loss of *Tle3* or *Tle4*, compared to control. While the transcript levels of all three TEAD targets increased in *Tle3* knockout cells, in *Tle4* knockout cells *Ankrd1* and *Cyr61* were decreased and *Ctgf* increased. *N* ≥ 3 set of cells, each qPCR was performed ≥three times in duplicate for each set of cells.

To study the neuromuscular phenotype resulting from the knockout of canonical Wnt signaling regulators, which expression is elevated in primary myotubes in response to AGRN, we generated bi-allelic primary knockout muscle cells for *Tle3* and *Tle4* genes using CRISPR/Cas9-mediated gene editing. To achieve an efficient gene knockout, guide sequences were designed to target the coding sequence closest to the start codon of the gene of interest. This approach was taken to ensure that as many splice variants as possible were affected. The guide sequences were then cloned into the pX330-U6-Chimeric_BB-CBh-hSpCas9 vector as described before (Cong et al., 2013). The guide sequences for *Tle3* were located in exon 4, while those for *Tle4* were located in exon 3 (Supplementary Figure S1). After co-transfecting the respective vectors and a GFP expressing plasmid into purified primary wild type muscle satellite cells with a low passage number (less than 3), single cells were sorted using FACS and clonally expanded. They were then screened for the absence of the protein of interest by western blot (Figure 5A) and immunofluorescence microscopy of cultured CRISPR knockout muscle cells (Figure 5B). Cells that were transfected with an empty pX330 plasmid and sorted using FACS were used as controls. The DNA regions surrounding the recombination site of each clone were sequenced to confirm and identify the genomic bi-allelic mutation (Supplementary Figure S1). The Degenerate Sequence Decoding strategy was used to decode the sequences of each gene variant from overlapping peaks of sequencing chromatograms (Ma et al., 2015). Only clones that frameshift mutations with premature stops on both alleles (Supplementary Figure S1) were used for further studies.

To investigate the ability of the *Tle3* and *Tle4* CRISPR knockout cells to form CHRN clusters in response to neural AGRN, several independent CRISPR knockout clones were analyzed. Equal numbers of respective cells were plated, differentiated for 5 days, and subsequently incubated with AGRN-conditioned media for 16 h to induce clustering of CHRNs. The CHRN clusters were visualized with rhodamine-coupled alpha-bungarotoxin (BTX) and cell nuclei were visualized with DAPI (Figure 5C). Interestingly, we observed a slight but significant decrease in AGRN-induced CHRN clustering in *Tle3* CRISPR knockout myotubes, while *Tle4* knockout myotubes showed a significant impairment of AGRN-induced CHRN clustering, as evidenced by a significant reduction in normalized total fluorescence intensity (Figure 5D). Subsequently, we investigated how the absence of *Tle3* or *Tle4* affects CHRN clustering. The expression of genes encoding the *Chrna1* and *Chrng* subunits, *Musk*, and *Dok7* was quantified in differentiated *Tle3* or *Tle4* CRISPR knockout primary muscle cells (Figure 5E). *Tle3* knockout resulted in a minor reduction in the expression of genes encoding the *Chrng* subunit and *Dok7*, while *Tle4* knockout led to a significant reduction in the expression of *Chrna1* and *Chrng* subunits as well as *Musk* (Figure 5E). Overall, *Tle4* knockout muscle cells showed the strongest reduction in synaptic gene expression in comparison to control. This reduction was also reflected in their inhibited ability to cluster CHRN induced by AGRN (Figures 5C,D). Therefore, the reduced amount of AGRN-induced CHRN clustering in the CRISPR knockout cells (Figures 5C,D) is at least partly due to the decreased expression of the involved synaptic genes (Figure 5E). To investigate the effect of *Tle3* or *Tle4* absence on the transcription of TCF/LEF target genes, we analyzed the transcript levels of the hallmark target gene *Axin2* in the respective CRISPR knockout cells. *Axin2* transcript amount was significantly reduced in *Tle3* knockout cells, but significantly increased in *Tle4* knockout cells (Figure 5F). Previously, a cross-link between canonical Wnt and Hippo signaling pathway members was reported in muscle fibers (Huraskin et al., 2016). The transcriptional co-activators YAP1 and TAZ are believed to be part of the CTNNB1-containing destruction complex of canonical Wnt signaling (Azzolin et al., 2012, 2014; Huraskin et al., 2016). Notably, TEAD target genes *Ankrd1*, *Cyr61* and *Ctgf* were found to have elevated transcription in *Tle3* knockout muscle cells (Figure 5G), while the expression of TEAD targets was differentially affected in *Tle4* knockout cells (Figure 5G).

## Discussion

In our investigation of transcriptional regulators implicated in CHRN clustering in muscle cells, we have identified *Tle3* and *Tle4* as potential CTNNB1-dependent transcriptional repressors. These repressors are expressed and localized in the nuclei of differentiated primary muscle cells (Figures 3C, 4D,E). Previously, TLE3 was reported to increase during differentiation in floating muscle fibers *ex vivo* and to repress the activity of MYOD1 which leads to suppressed myogenesis (Kokabu et al., 2017; Kumar et al., 2023). TLE3 was also found to be involved in the regulation of *Myh3* transcription (Kumar et al., 2023). The suggestion that TLE3 serves as a dual-function switch, driving the formation of both active and repressive transcriptional complexes that facilitate the adipogenic program, is noteworthy (Villanueva et al., 2011). This points to the potential direct or indirect capability of TLE3 to activate transcription in a tissue-specific manner. TLE4 expression decreases during myogenesis but subsequently increases during differentiation (Agarwal et al., 2022). TLE4 represses PAX7-mediated *Myf5* transcription, thus maintaining satellite cell quiescence (Agarwal et al., 2022). Whether TLE3 and/or TLE4 are involved in postsynaptic transcription has never been investigated.

The lack of β-galactosidase reporter expression in Axin2^+/lacZ^ muscles after denervation indicates that an active nerve is necessary to initiate the induction of canonical Wnt signaling in adult muscle fibers (Figure 1). There may be several mechanisms responsible for this phenomenon. One possibility is that the motor neuron secretes canonical WNTs at the nerve terminal, which subsequently activates postsynaptic CTNNB1-dependent signaling. Previous reports suggest that WNTs are secreted by the nerve at the NMJ to promote CHRN clustering (Henriquez et al., 2008; Jing et al., 2009; Shen et al., 2018). However, the reported effects are believed to be exerted through non-canonical Wnt pathways or AGRN/LRP4/MUSK signaling. In contrast, muscle cells release both canonical and non-canonical WNTs (Wang et al., 2008; Strochlic et al., 2012; Zhang et al., 2012; Shen et al., 2018). The secretion of WNT from muscle cells is maybe regulated by synaptic activity as observed in the CNS (Chen et al., 2006; Li et al., 2012) and at the *Drosophila* NMJ (Ataman et al., 2008). However, it should be noted that the loss of WNT secretion regulator Wingless in the muscle had no effect on the neuromuscular phenotype (Remedio et al., 2016; Shen et al., 2018). It is possible that secreted WNT inhibitors are involved, as the expression of the soluble WNT inhibitor SFRP1 was upregulated at the NMJs in denervated muscle (Svensson et al., 2008). Our lab has previously demonstrated that differentiation of muscle cells results in the suppression of canonical Wnt signaling activity by DKK1 (dickkopf 1) (Huraskin et al., 2016; Gessler et al., 2022).

Moreover, the denervation-mediated loss of *Axin2* expression (Figures 1A,B) is linked with reduced transcript levels of *Axin1* (Figure 1B), *Tcf7l1* (Figure 1D), *Tle1*, *Tle2, Tle4* (Figure 1E), and *Ctnnb1* (Figure 1C). The reduction of *Axin1* transcript level is rather surprising, because *Axin1* is believed to be mostly ubiquitously and constitutively expressed (Dr. Frank Costantini, Columbia University, United States, personal communication). Our findings indicate that denervation not only turns off canonical Wnt signaling but also triggers complex alterations in the expression of various positive and negative regulators of Wnt signaling. In other contexts, these types of expression changes have been repeatedly associated with cross-regulation involving TGF-β/SMAD signaling (Dao et al., 2007; Guo et al., 2008; Zhang and Dressler, 2013; Biressi et al., 2014; Gillespie et al., 2018). TGF-β/SMAD signaling is active at the NMJ (Jiang et al., 2000; McLennan and Koishi, 2002) and is upregulated after denervation (McLennan et al., 1998). Denervation may be linked to alterations in TGF-β signaling activity, as evidenced by the concurrent decrease of *Axin1* and *Axin2* expression (Figures 1A,B) and other regulators of canonical Wnt signaling, like the TLE repressors (Figure 1E). Of note, members of the Hippo pathway, YAP1, TAZ, TEAD1 and TEAD4, have been reported to be important in regulating postsynaptic gene expression (Zhao et al., 2017; Gessler et al., 2023); not to mention that this may occur in a concerted manner through crosstalk with canonical Wnt signaling (Huraskin et al., 2016; Gessler et al., 2023). Negative feedback mechanisms in myogenesis regulate myotube formation by increasing CTNNB1-dependent *Axin2* expression and YAP1/TAZ-TEAD signaling activity in response to canonical Wnt (Huraskin et al., 2016). Previous reports suggest that the Wnt and YAP/TAZ-TEAD pathways have similar effects in regulating CHRN clustering. On the one hand, the canonical and non-canonical Wnt pathways play opposing roles at the NMJ, but on the other hand, they work together to regulate the assembly and maintenance of the postsynaptic apparatus through anterograde and retrograde signaling (Cisternas et al., 2014). In this context, the TLE transcriptional repressors TLE3 and TLE4 are essential elements of regulation. In contrast, YAP1 and TAZ, transcriptional co-activators of the Hippo pathway, work together with AGRN/MUSK/LRP4 signaling to form and regenerate NMJs (Zhao et al., 2017).

Additionally, our findings indicate that TLE transcription was increased in neural AGRN-treated primary myotubes (Figure 4C). The significance of these repressors in the postsynapse was evident in *Tle4* knockout primary myotubes and, to a lower degree, in *Tle3* knockout primary myotubes (Figure 5). These cells formed fewer CHRN clusters than control cells when exposed to AGRN, resulting in reduced BTX staining (Figures 5C,D). The suppression of CHRN clusters is increased in *Tle4* knockout cells, which is accompanied by a decrease in the expression of synaptic genes (Figure 5E). Surprisingly, *Chrng* transcript level is downregulated in the absence of *Tle3* or *Tle4* (Figure 5E), while denervation stimulates *Chrng* transcription which is accompanied by lower *Tle3* and *Tle4* transcript levels (Figure 1B). The down-regulation of *Chrng* transcript level in *Tle3* or *Tle4* knockout cells is not linked to any cell survival impairments (data not shown). Future studies should help to understand whether transcript levels of other key players of canonical Wnt or Hippo signaling are modified in the absence of *Tle3* or *Tle4*. *Tle* transcriptional repressors act as transcriptional corepressors of TCF/LEF mediated transcription in the absence of *Ctnnb1* in the Wnt OFF state (Brantjes et al., 2001). Therefore, their loss, as well as the effect of increasing muscular *Ctnnb1* gain-of-function (Wang et al., 2008; Wu et al., 2012), appears to have a detrimental effect on the NMJ. At this step, it is not possible to rule out a CHRN clustering defect, but the reduced transcript levels of *Chrn* genes argue towards transcriptional regulation (Figure 5E). However, the divergent alterations of *Axin2* expression pattern (Figure 5F) and TEAD target genes (Figure 5G) in the absence of *Tle3* and *Tle4* suggest distinct mechanisms of action.

The reduction in canonical Wnt signaling activity, likely due to the involvement of TLE3 and TLE4, along with the reported simultaneous increase in *Yap1*/*Taz-Tead* expression and activity (Gessler et al., 2023), observed immediately after denervation could potentially be a physiological response that maximizes positive input for promoting reinnervation and synaptic gene expression while counteracting denervation-induced muscle atrophy.

## Data availability statement

The original contributions presented in the study are included in the article/Supplementary material, further inquiries can be directed to the corresponding author.

## Ethics statement

Mouse experiments were performed in accordance with animal welfare laws and approved by the responsible local committees (animal protection officer, Sachgebiet Tierschutzangelegenheiten, FAU Erlangen-Nürnberg, AZ: I/39/EE006 and TS-07/11), government bodies (Regierung von Unterfranken). The study was conducted in accordance with the local legislation and institutional requirements.

## Author contributions

LG: Writing – review & editing, Visualization, Validation, Supervision, Software, Resources, Methodology, Investigation, Formal analysis, Data curation, Conceptualization. DH: Writing – review & editing, Visualization, Validation, Supervision, Software, Resources, Methodology, Investigation, Formal analysis, Data curation, Conceptualization. NE: Writing – review & editing, Visualization, Validation, Supervision, Software, Resources, Methodology, Investigation, Formal analysis, Data curation, Conceptualization. SH: Writing – review & editing, Writing – original draft, Visualization, Validation, Supervision, Software, Resources, Project administration, Methodology, Investigation, Funding acquisition, Formal analysis, Data curation, Conceptualization.

## Footnotes

^1^ https://fiji.sc/
