# Supplementary material for: The impact of canonical Wnt transcriptional repressors TLE3 and TLE4 on postsynaptic transcription at the neuromuscular junction
Source: Front Mol Neurosci. 2024 Mar 27;17:1360368. doi: 10.3389/fnmol.2024.1360368 (PMC11004254; doi:10.3389/fnmol.2024.1360368)
Supplement: Supplementary file 1 [file Data_Sheet_1.docx]

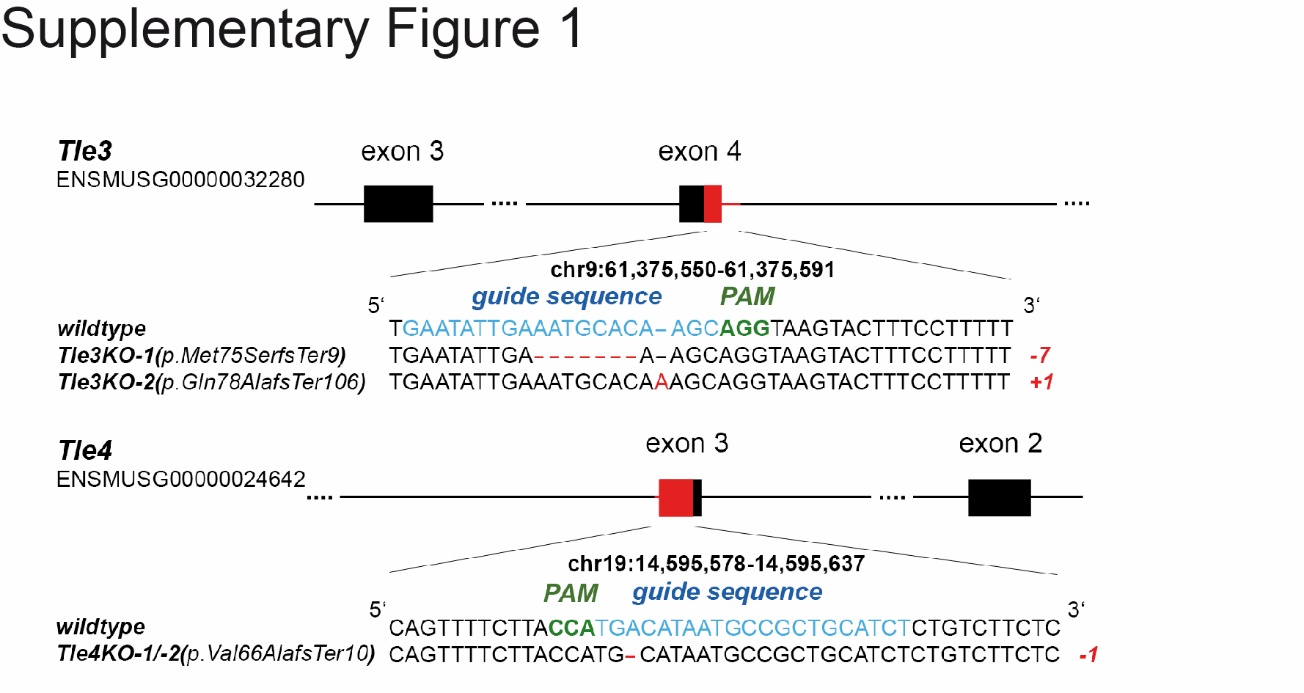


**Supplementary figure 1: Generation and validation of primary muscle cells with knocked out *Tle3* and *Tle4* genes via CRISPR/Cas9 genome editing.**

For CRISPR/Cas9 mediated knockout of the genes of interest optimal 20bp guide sequences (blue) adjacent to 3-mer PAM sequence NGG (green) were chosen in one of the early coding exons to favor frameshift mutations that would result in a premature stop and nonsense mediated decay of target peptides. The actual indels in any of the clones used in this study, as confirmed by genomic sequencing, are shown in red for each allele and compared against the wild type genomic sequence. The resulting protein variants are designated in parentheses according to recommendations of the Human Genome Variation Society. The genomic coordinates of the presented sequences are based on the Mouse July 2007 (NCBI37/mm9) genome assembly.

**Supplementary Table S1**

Tabular presentation of oligonucleotide sequences

| **Genomic target** | **Orientation** | **Sequence** |
| --- | --- | --- |
| Tle3 target sequence for cloning into pX330 | forward | CACCGAATATTGAAATGCACAAGC |
|  | reverse | AAACGCTTGTGCATTTCAATATTC |
| Tle4 target sequence for cloning into pX330 | forward | CACCGATGCAGCGGCATTATGTCA |
|  | reverse | AAACTGACATAATGCCGCTGCATC |
| pX330 sequencing |  | GGCCTATTTCCCATGATTCCTTC |
| Tle3 amplification and sequencing of CRISPR/Cas9 target site | forward | GTTAAAGCCTTGTGAGGGGGA |
|  | reverse | TCCTTCAAGACCAAGTGGGAAT |
| Tle4 amplification and sequencing of CRISPR/Cas9 target site | forward | CTTGCTTTCAGTCTCCCAAGC |
|  | reverse | AGAGGAAACGACGTGGATTTGG |
| Pax7 qPCR | forward | GCTACCAGTACAGCCAGTATG |
|  | reverse | GTCACTAAGCATGGGTAGATG |
| Myod1 qPCR | forward | TGGCATGATGGATTACAGCGG |
|  | reverse | GGTCTGGGTTCCCTGTTCTG |
| Myog qPCR | forward | CAGTACATTGAGCGCCTACA |
|  | reverse | GCCTGACAGACAATCTCAGT |
| Myh3 qPCR | forward | ACCCCAGGGGCCATGGAACA |
|  | reverse | CTCCGCGCTCTTCAGCAGGG |
| Tle1 qPCR and In situ riboprobes | forward | ATGGACCCCCAGTACCTCTC |
|  | reverse | TGGAGAAGAAGGGTCCTCGT |
| Tle2 qPCR and In situ riboprobes | forward | TCAGTACCACAGCCTCAAGC |
|  | reverse | CAGGAGGCTGTTCAGTTCCC |
| Tle3 qPCR and In situ riboprobes | forward | GGTACGTGGACTCCCCAATC |
|  | reverse | CACGGAATTGTTCGTGCTGG |
| Tle4 qPCR and In situ riboprobes | forward | AGCAAGATGTACCCGCAGAC |
|  | reverse | GAGTTGTTGCTGCCCAATGA |
| Tcf7 qPCR | forward | CGAGTACATGGAGAAGCCGAG |
|  | reverse | CGGCCTGTGAACTCCTTGCT |
| Tcf7l1 qPCR | forward | TCAGAGCAGTAGCTCGGACT |
|  | reverse | TGTTGGACAAGTGTGCTGGA |
| Tcf7l2 qPCR | forward | GGAGAGAAAGACCCCAAAGCA |
|  | reverse | TCTTTCCGCCTCGGAATCG |
| Lef1 qPCR | forward | ACAGATCACCCCACCCATTG |
|  | reverse | TTCTGGGACCTGTACCTGAAGT |
| Musk qPCR | forward | GCCTTGGTTGAAGAAGTAGC |
|  | reverse | CTTGATCCAGGACACAGATG |
| Dok7 qPCR | forward | GAATTCGGTTCTCTGCTCAGTCTG |
|  | reverse | CCAAGTCCATGTAGTGCAGCTG |
| Chrna1 qPCR and In situ riboprobes | forward | ACGCTGAGCATCTCTGTCTT |
|  | reverse | TTGGACTCCTGGTCTGACTT |
| Chrng qPCR | forward | GGTCAATGTCAGCCTGAAGC |
|  | reverse | GCACATGCATCCGTAACAGC |
| Ctnnb1 qPCR | forward | TCTGGAATCCATTCTGGTGC |
|  | reverse | CTCATCTAGCGTCTCAGGGA |
| Axin1 qPCR | forward | TGCAGTGGATCATTGAGGGAG |
|  | reverse | TTTTGTCCTCTGCTTGGAGGG |
| Axin2 qPCR | forward | GACGGACAGTAGCGTAGATGG |
|  | reverse | GGGTCCTCTTCATAGCTGCC |
| Cyr61 qPCR | forward | AAGAGGCTTCCTGTCTTTGGC |
|  | reverse | ATCGGAACCGCATCTTCACA |
| Ctgf qPCR | forward | CTAGCTGCCTACCGACTGGAA |
|  | reverse | CAAACTTGACAGGCTTGGCG |
| Ankrd1 qPCR | forward | TGGAGGAAACGCAGATGTCC |
|  | reverse | TCCCAGCACAGTTCTTGACC |
| Rpl8 qPCR | forward | GTTCGTGTACTGCGGCAAGA |
|  | reverse | ACAGGATTCATGGCCACACC |
